# (*m*-Phenyl­enedimethyl­ene)diammonium dichloride

**DOI:** 10.1107/S1600536808031334

**Published:** 2008-10-04

**Authors:** Hua Cheng, Huisheng Li

**Affiliations:** a Department of Chemistry and Biology, Xiangfan University, Xiangfan 441053, People’s Republic of China

## Abstract

The asymmetric unit of the title compound, C_8_H_14_N_2_^2+^·2Cl^−^, contains one and a half of the dications and three chloride anions. The half molecule is completed by crystallographic twofold symmetry with two C atoms lying on the rotation axis. The two ammonium groups in each cation adopt a *trans* conformation with respect ot the benzene ring. The ammonium groups and chloride anions are involved in the formation of a three-dimensional N—H⋯Cl hydrogen-bonding network, which stabilizes the crystal packing.

## Related literature

For general background and applications, see: Pasini & Zunino (1987 ▶); Otsuka *et al.* (1990 ▶); Michalson & Smuszkovicz (1989 ▶); Reedijk (1996 ▶); Blaser (1992 ▶); Soai & Niwa (1992 ▶); Jacobsen (1993 ▶); Kolb *et al.* (1994 ▶).
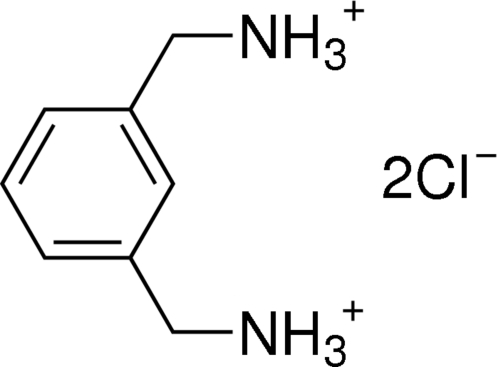


## Experimental

### 

#### Crystal data

C_8_H_14_N_2_^2+^·2Cl^−^
*M* *_r_* = 209.11
Monoclinic, 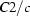

*a* = 27.5859 (18) Å
*b* = 13.1594 (14) Å
*c* = 8.8324 (6) Å
β = 103.539 (1)°
*V* = 3117.2 (4) Å^3^
*Z* = 12
Mo *K*α radiation
μ = 0.58 mm^−1^
*T* = 298 (2) K
0.20 × 0.10 × 0.10 mm

#### Data collection

Bruker SMART CCD area-detector diffractometer
Absorption correction: multi-scan (*SADABS*; Sheldrick, 1997 ▶) *T*_min_ = 0.893, *T*_max_ = 0.945
14623 measured reflections
3066 independent reflections
2615 reflections with *I* > 2σ(*I*)
*R*_int_ = 0.103

#### Refinement

*R*[*F* ^2^ > 2σ(*F* ^2^)] = 0.043
*wR*(*F* ^2^) = 0.118
*S* = 1.06
3066 reflections
191 parameters
9 restraints
H atoms treated by a mixture of independent and constrained refinement
Δρ_max_ = 0.36 e Å^−3^
Δρ_min_ = −0.31 e Å^−3^

### 

Data collection: *SMART* (Bruker, 2001 ▶); cell refinement: *SAINT* (Bruker, 1999 ▶); data reduction: *SAINT*; program(s) used to solve structure: *SHELXS97* (Sheldrick, 2008 ▶); program(s) used to refine structure: *SHELXL97* (Sheldrick, 2008 ▶); molecular graphics: *SHELXTL* (Sheldrick, 2008 ▶); software used to prepare material for publication: *SHELXTL*.

## Supplementary Material

Crystal structure: contains datablocks I, global. DOI: 10.1107/S1600536808031334/cv2456sup1.cif

Structure factors: contains datablocks I. DOI: 10.1107/S1600536808031334/cv2456Isup2.hkl

Additional supplementary materials:  crystallographic information; 3D view; checkCIF report

## Figures and Tables

**Table 1:** Hydrogen-bond geometry (Å, °)

*D*—H⋯*A*	*D*—H	H⋯*A*	*D*⋯*A*	*D*—H⋯*A*
N3—H3*B*⋯Cl1	0.878 (16)	2.338 (16)	3.206 (2)	170 (2)
N2—H2*C*⋯Cl3	0.927 (16)	2.360 (17)	3.2453 (19)	160 (2)
N1—H1*B*⋯Cl2	0.869 (15)	2.276 (17)	3.1186 (18)	163 (2)
N3—H3*C*⋯Cl2^i^	0.880 (17)	2.343 (19)	3.171 (2)	157 (2)
N2—H2*B*⋯Cl1^ii^	0.860 (16)	2.58 (2)	3.189 (2)	129.0 (19)
N1—H1*C*⋯Cl3^iii^	0.884 (15)	2.341 (17)	3.2071 (18)	166 (2)
N2—H2*A*⋯Cl2^iv^	0.842 (16)	2.442 (18)	3.201 (2)	150 (2)
N1—H1*A*⋯Cl3^iv^	0.858 (16)	2.506 (18)	3.2527 (17)	146.0 (19)

Symmetry codes: (i) 
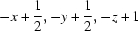
; (ii) 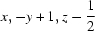; (iii) 
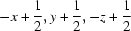
; (iv) 
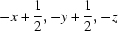
.

**Fig. 1.**
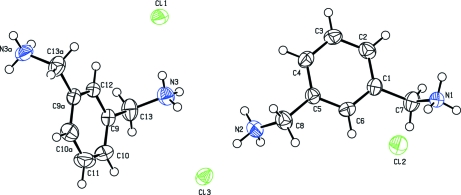
View of (I) showing the atom-labelling scheme. Displacement ellipsoids are drawn at the 30% probability level. H atoms are represented by spheres of arbitrary radius.

## supplementary crystallographic information

### Comment

The diamine compounds are important in biologically active natural products (Pasini & Zunino, 1987; Otsuka *et al.*, 1990), in medicinal chemistry (Michalson & Smuszkovicz, 1989; Reedijk, 1996). They are also used as chiral auxiliaries and chiral ligands in asymmetric catalysis (Blaser, 1992; Soai & Niwa, 1992; Jacobsen, 1993; Kolb *et al.*, 1994). Herewith we present the title diamine compound, (I).

In (I) (Fig. 1), all bond lengths and angles are normal. Two amino groups in the dications adopt trans-conformation and each amino group form three N—H···Cl hydrogen bonds (Table 1) to stabilize the crystal packing.

### Experimental

1,3-Phenylenedimethanamine was dissolved in ethanol, then 1N HCl was dropped to the solution. Colourless, block-like crystals of (I) suitable for X-ray data collection were obtained by slow evaporation of ethanol at 283 K.

### Refinement

All H atoms were initially located in a difference Fourier map. C-bound H atoms were placed in idealized positions (C—H = 0.93–0.97 Å) and refined as riding, with U_iso_(H) = 1.2U_eq_(C). Amino H atoms were refined with bond restraint of N—H = 0.88 (3) Å and constrained displacement parameter U_iso_(H) = 1.2U_eq_(N).

### Crystal data

**Table:**

C_8_H_14_N_2_^2+^·2Cl^−^	*F*(000) = 1320
*M**_r_* = 209.11	*D*_x_ = 1.337 Mg m^−^^3^
Monoclinic, *C*2/*c*	Mo *K*α radiation, λ = 0.71073 Å
Hall symbol: -c/2yc	Cell parameters from 6157 reflections
*a* = 27.5859 (18) Å	θ = 2.8–27.8°
*b* = 13.1594 (14) Å	µ = 0.58 mm^−^^1^
*c* = 8.8324 (6) Å	*T* = 298 K
β = 103.539 (1)°	Block, colourless
*V* = 3117.2 (4) Å^3^	0.20 × 0.10 × 0.10 mm
*Z* = 12	

### Data collection

**Table:**

Bruker SMART CCD area-detector diffractometer	3066 independent reflections
Radiation source: fine-focus sealed tube	2615 reflections with *I* > 2σ(*I*)
graphite	*R*_int_ = 0.103
φ and ω scans	θ_max_ = 26.0°, θ_min_ = 1.5°
Absorption correction: multi-scan (SADABS; Sheldrick, 1997)	*h* = −33→33
*T*_min_ = 0.894, *T*_max_ = 0.945	*k* = −14→16
14623 measured reflections	*l* = −10→10

### Refinement

**Table:**

Refinement on *F*^2^	Primary atom site location: structure-invariant direct methods
Least-squares matrix: full	Secondary atom site location: difference Fourier map
*R*[*F*^2^ > 2σ(*F*^2^)] = 0.043	Hydrogen site location: inferred from neighbouring sites
*wR*(*F*^2^) = 0.118	H atoms treated by a mixture of independent and constrained refinement
*S* = 1.06	*w* = 1/[σ^2^(*F*_o_^2^) + (0.0717*P*)^2^] where *P* = (*F*_o_^2^ + 2*F*_c_^2^)/3
3066 reflections	(Δ/σ)_max_ = 0.001
191 parameters	Δρ_max_ = 0.36 e Å^−^^3^
9 restraints	Δρ_min_ = −0.31 e Å^−^^3^

### Special details

**Table:**

Geometry. All esds (except the esd in the dihedral angle between two l.s. planes) are estimated using the full covariance matrix. The cell esds are taken into account individually in the estimation of esds in distances, angles and torsion angles; correlations between esds in cell parameters are only used when they are defined by crystal symmetry. An approximate (isotropic) treatment of cell esds is used for estimating esds involving l.s. planes.
Refinement. Refinement of F^2^ against ALL reflections. The weighted R-factor wR and goodness of fit S are based on F^2^, conventional R-factors R are based on F, with F set to zero for negative F^2^. The threshold expression of F^2^ > 2sigma(F^2^) is used only for calculating R-factors(gt) etc. and is not relevant to the choice of reflections for refinement. R-factors based on F^2^ are statistically about twice as large as those based on F, and R- factors based on ALL data will be even larger.

### Fractional atomic coordinates and isotropic or equivalent isotropic displacement parameters (Å^2^)

**Table:**

	*x*	*y*	*z*	*U*_iso_*/*U*_eq_	
C1	0.27771 (7)	0.50258 (14)	−0.0022 (2)	0.0355 (4)	
C2	0.26673 (7)	0.60114 (16)	0.0327 (2)	0.0414 (5)	
H2	0.2856	0.6547	0.0089	0.050*	
C3	0.22778 (7)	0.62046 (16)	0.1031 (2)	0.0438 (5)	
H3	0.2206	0.6870	0.1261	0.053*	
C4	0.19964 (7)	0.54155 (16)	0.1392 (2)	0.0398 (5)	
H4	0.1735	0.5549	0.1865	0.048*	
C5	0.21012 (6)	0.44213 (14)	0.1052 (2)	0.0341 (4)	
C6	0.24907 (7)	0.42332 (14)	0.0342 (2)	0.0355 (4)	
H6	0.2561	0.3569	0.0106	0.043*	
C7	0.31958 (8)	0.48207 (17)	−0.0814 (2)	0.0447 (5)	
H7A	0.3118	0.4215	−0.1450	0.054*	
H7B	0.3220	0.5384	−0.1500	0.054*	
C8	0.18156 (7)	0.35486 (16)	0.1535 (3)	0.0458 (5)	
H8A	0.1995	0.2922	0.1469	0.055*	
H8B	0.1802	0.3642	0.2613	0.055*	
C9	0.00722 (7)	0.23186 (15)	0.6209 (2)	0.0349 (4)	
C10	0.00730 (8)	0.12695 (16)	0.6221 (3)	0.0466 (5)	
H10	0.0123	0.0915	0.5360	0.056*	
C11	0.0000	0.0741 (2)	0.7500	0.0553 (8)	
H11	0.0000	0.0034	0.7500	0.066*	
C12	0.0000	0.2839 (2)	0.7500	0.0349 (6)	
H12	0.0000	0.3546	0.7500	0.042*	
C13	0.01564 (8)	0.28914 (19)	0.4806 (3)	0.0483 (5)	
H13A	0.0100	0.2439	0.3915	0.058*	
H13B	−0.0081	0.3446	0.4561	0.058*	
Cl1	0.08043 (2)	0.50782 (4)	0.76455 (6)	0.04504 (18)	
Cl2	0.35181 (2)	0.28315 (4)	0.23278 (6)	0.04852 (19)	
Cl3	0.094656 (19)	0.15614 (4)	0.24153 (6)	0.04295 (18)	
N1	0.36851 (6)	0.46791 (14)	0.0298 (2)	0.0375 (4)	
H1C	0.3787 (8)	0.5257 (13)	0.078 (3)	0.045*	
H1A	0.3894 (7)	0.4518 (17)	−0.025 (2)	0.045*	
H1B	0.3683 (8)	0.4230 (15)	0.102 (2)	0.045*	
N2	0.13028 (7)	0.34496 (14)	0.0574 (2)	0.0433 (4)	
H2A	0.1269 (9)	0.3278 (17)	−0.036 (2)	0.052*	
H2B	0.1123 (8)	0.3989 (15)	0.050 (3)	0.052*	
H2C	0.1129 (8)	0.2921 (15)	0.090 (3)	0.052*	
N3	0.06651 (7)	0.32989 (15)	0.5100 (2)	0.0433 (4)	
H3C	0.0906 (7)	0.2890 (16)	0.557 (3)	0.052*	
H3A	0.0729 (8)	0.3619 (17)	0.430 (2)	0.052*	
H3B	0.0690 (8)	0.3733 (16)	0.587 (2)	0.052*	

### Atomic displacement parameters (Å^2^)

**Table:**

	*U*^11^	*U*^22^	*U*^33^	*U*^12^	*U*^13^	*U*^23^
C1	0.0337 (10)	0.0412 (11)	0.0320 (10)	0.0014 (8)	0.0086 (8)	0.0026 (8)
C2	0.0419 (11)	0.0340 (11)	0.0477 (11)	−0.0020 (9)	0.0093 (9)	0.0080 (9)
C3	0.0437 (11)	0.0300 (11)	0.0564 (13)	0.0065 (9)	0.0093 (10)	0.0010 (9)
C4	0.0348 (10)	0.0394 (11)	0.0465 (11)	0.0074 (8)	0.0121 (9)	0.0002 (9)
C5	0.0312 (9)	0.0350 (11)	0.0351 (10)	−0.0009 (8)	0.0060 (8)	0.0031 (8)
C6	0.0383 (10)	0.0304 (10)	0.0373 (10)	0.0049 (8)	0.0080 (8)	−0.0016 (8)
C7	0.0441 (12)	0.0572 (14)	0.0357 (11)	0.0029 (10)	0.0155 (10)	0.0038 (9)
C8	0.0410 (11)	0.0424 (12)	0.0537 (13)	−0.0015 (9)	0.0106 (10)	0.0110 (10)
C9	0.0297 (9)	0.0420 (12)	0.0345 (10)	−0.0004 (8)	0.0103 (8)	0.0021 (8)
C10	0.0534 (12)	0.0404 (13)	0.0487 (13)	0.0014 (10)	0.0174 (10)	−0.0118 (10)
C11	0.070 (2)	0.0306 (16)	0.067 (2)	0.000	0.0181 (18)	0.000
C12	0.0321 (13)	0.0293 (14)	0.0460 (16)	0.000	0.0148 (12)	0.000
C13	0.0415 (11)	0.0678 (16)	0.0373 (11)	−0.0028 (10)	0.0126 (9)	0.0077 (10)
Cl1	0.0547 (3)	0.0370 (3)	0.0492 (3)	−0.0046 (2)	0.0237 (3)	−0.0082 (2)
Cl2	0.0507 (3)	0.0449 (3)	0.0514 (3)	0.0066 (2)	0.0148 (3)	0.0119 (2)
Cl3	0.0483 (3)	0.0368 (3)	0.0480 (3)	0.0090 (2)	0.0199 (3)	0.0070 (2)
N1	0.0371 (9)	0.0345 (9)	0.0450 (10)	−0.0037 (7)	0.0179 (8)	−0.0048 (7)
N2	0.0414 (10)	0.0364 (10)	0.0530 (11)	−0.0058 (8)	0.0132 (9)	0.0012 (8)
N3	0.0455 (10)	0.0417 (11)	0.0438 (11)	−0.0037 (8)	0.0130 (9)	0.0101 (8)

### Geometric parameters (Å, °)

**Table:**

C1—C2	1.383 (3)	C9—C13	1.514 (3)
C1—C6	1.391 (3)	C10—C11	1.381 (3)
C1—C7	1.508 (3)	C10—H10	0.9300
C2—C3	1.385 (3)	C11—C10^i^	1.381 (3)
C2—H2	0.9300	C11—H11	0.9300
C3—C4	1.378 (3)	C12—C9^i^	1.384 (2)
C3—H3	0.9300	C12—H12	0.9300
C4—C5	1.388 (3)	C13—N3	1.468 (3)
C4—H4	0.9300	C13—H13A	0.9700
C5—C6	1.387 (2)	C13—H13B	0.9700
C5—C8	1.510 (3)	N1—H1C	0.884 (15)
C6—H6	0.9300	N1—H1A	0.858 (16)
C7—N1	1.483 (3)	N1—H1B	0.869 (15)
C7—H7A	0.9700	N2—H2A	0.842 (16)
C7—H7B	0.9700	N2—H2B	0.860 (16)
C8—N2	1.475 (3)	N2—H2C	0.927 (16)
C8—H8A	0.9700	N3—H3C	0.880 (17)
C8—H8B	0.9700	N3—H3A	0.877 (16)
C9—C10	1.381 (3)	N3—H3B	0.878 (16)
C9—C12	1.384 (2)		
			
C2—C1—C6	119.05 (17)	C9—C10—C11	120.7 (2)
C2—C1—C7	120.19 (17)	C9—C10—H10	119.7
C6—C1—C7	120.75 (17)	C11—C10—H10	119.7
C1—C2—C3	120.39 (18)	C10—C11—C10^i^	119.5 (3)
C1—C2—H2	119.8	C10—C11—H11	120.2
C3—C2—H2	119.8	C10^i^—C11—H11	120.2
C4—C3—C2	120.25 (19)	C9—C12—C9^i^	120.6 (3)
C4—C3—H3	119.9	C9—C12—H12	119.7
C2—C3—H3	119.9	C9^i^—C12—H12	119.7
C3—C4—C5	120.21 (17)	N3—C13—C9	111.14 (18)
C3—C4—H4	119.9	N3—C13—H13A	109.4
C5—C4—H4	119.9	C9—C13—H13A	109.4
C6—C5—C4	119.26 (17)	N3—C13—H13B	109.4
C6—C5—C8	120.17 (17)	C9—C13—H13B	109.4
C4—C5—C8	120.46 (17)	H13A—C13—H13B	108.0
C5—C6—C1	120.85 (17)	C7—N1—H1C	110.3 (15)
C5—C6—H6	119.6	C7—N1—H1A	106.6 (15)
C1—C6—H6	119.6	H1C—N1—H1A	108 (2)
N1—C7—C1	113.13 (16)	C7—N1—H1B	114.1 (14)
N1—C7—H7A	109.0	H1C—N1—H1B	107 (2)
C1—C7—H7A	109.0	H1A—N1—H1B	111 (2)
N1—C7—H7B	109.0	C8—N2—H2A	117.4 (18)
C1—C7—H7B	109.0	C8—N2—H2B	115.4 (16)
H7A—C7—H7B	107.8	H2A—N2—H2B	103 (2)
N2—C8—C5	113.48 (17)	C8—N2—H2C	112.6 (15)
N2—C8—H8A	108.9	H2A—N2—H2C	99 (2)
C5—C8—H8A	108.9	H2B—N2—H2C	108 (2)
N2—C8—H8B	108.9	C13—N3—H3C	116.4 (16)
C5—C8—H8B	108.9	C13—N3—H3A	113.4 (16)
H8A—C8—H8B	107.7	H3C—N3—H3A	114 (2)
C10—C9—C12	119.27 (18)	C13—N3—H3B	105.8 (15)
C10—C9—C13	120.27 (18)	H3C—N3—H3B	97 (2)
C12—C9—C13	120.5 (2)	H3A—N3—H3B	109 (2)
			
C6—C1—C2—C3	0.0 (3)	C6—C1—C7—N1	−92.2 (2)
C7—C1—C2—C3	178.93 (19)	C6—C5—C8—N2	−109.5 (2)
C1—C2—C3—C4	0.1 (3)	C4—C5—C8—N2	74.2 (2)
C2—C3—C4—C5	0.0 (3)	C12—C9—C10—C11	0.4 (3)
C3—C4—C5—C6	−0.2 (3)	C13—C9—C10—C11	179.56 (17)
C3—C4—C5—C8	176.14 (19)	C9—C10—C11—C10^i^	−0.18 (13)
C4—C5—C6—C1	0.4 (3)	C10—C9—C12—C9^i^	−0.18 (13)
C8—C5—C6—C1	−175.99 (18)	C13—C9—C12—C9^i^	−179.4 (2)
C2—C1—C6—C5	−0.3 (3)	C10—C9—C13—N3	−103.1 (2)
C7—C1—C6—C5	−179.17 (18)	C12—C9—C13—N3	76.1 (2)
C2—C1—C7—N1	88.9 (2)		

Symmetry codes: (i) −*x*, *y*, −*z*+3/2.

### Hydrogen-bond geometry (Å, °)

**Table:**

*D*—H···*A*	*D*—H	H···*A*	*D*···*A*	*D*—H···*A*
N3—H3B···Cl1	0.88 (2)	2.34 (2)	3.206 (2)	170 (2)
N2—H2C···Cl3	0.93 (2)	2.36 (2)	3.2453 (19)	160 (2)
N1—H1B···Cl2	0.87 (2)	2.28 (2)	3.1186 (18)	163 (2)
N3—H3C···Cl2^ii^	0.88 (2)	2.34 (2)	3.171 (2)	157 (2)
N2—H2B···Cl1^iii^	0.86 (2)	2.58 (2)	3.189 (2)	129 (2)
N1—H1C···Cl3^iv^	0.88 (2)	2.34 (2)	3.2071 (18)	166 (2)
N2—H2A···Cl2^v^	0.84 (2)	2.44 (2)	3.201 (2)	150 (2)
N1—H1A···Cl3^v^	0.86 (2)	2.51 (2)	3.2527 (17)	146 (2)

Symmetry codes: (ii) −*x*+1/2, −*y*+1/2, −*z*+1; (iii) *x*, −*y*+1, *z*−1/2; (iv) −*x*+1/2, *y*+1/2, −*z*+1/2; (v) −*x*+1/2, −*y*+1/2, −*z*.
